# The karyotype of three Brazilian Terrarana frogs (Amphibia, Anura) with evidence of a new *Barycholos* species

**DOI:** 10.1590/S1415-47572009005000044

**Published:** 2009-09-01

**Authors:** Sérgio Siqueira, Odair Aguiar, André Pansonato, Ariovaldo A. Giaretta, Christine Strüssmann, Itamar Martins, Shirlei M. Recco-Pimentel

**Affiliations:** 1 Departamento de Biologia Celular, Instituto de Biologia, Universidade Estadual de Campinas, Campinas, SPBrazil; 2 Departamento de Biociências, Universidade Federal de São Paulo, Santos, SPBrazil; 3 Programa de Pós-Graduação em Ecologia e Conservação da Biodiversidade, Universidade Federal de Mato Grosso, Cuiabá, MTBrazil; 4 Departamento de Biociências, Instituto de Biologia, Universidade Federal de Uberlândia, Uberlândia, MGBrazil; 5 Departamento de Ciências Básicas e Produção Animal, Universidade Federal de Mato Grosso, Cuiabá, MTBrazil; 6 Departamento de Biologia, Universidade de Taubaté, Taubaté, SPBrazil

**Keywords:** cytogenetics, *Barycholos*, *Ischnocnema*, *Pristimantis*, Ag-NOR, C-banding

## Abstract

A recent substantial rearrangement of the 882 described eleutherodactyline frog species has considerably improved the understanding of their systematics. Nevertheless, many taxonomic aspects of the South American eleutherodactyline species remain unknown and require further investigation using morphological, cytogenetic and molecular approaches. In this work, the karyotypes of the Brazilian species *Ischnocnema juipoca* (Atibaia and Campos do Jordão, SP)*, Barycholos* cf. *ternetzi* (Uberlândia, MG, and Porto Nacional, TO), and *Pristimantis crepitans* (Chapada dos Guimarães and São Vicente, MT) were analyzed using Giemsa staining, Ag-NOR labeling, and C-banding techniques. All individuals had a diploid number of 22 chromosomes, but the Fundamental Numbers were different among species. The herein described low chromosome number of *Pristimantis crepitans* is unique within this genus, suggesting that cytogenetically this species is not closely related either to its congeneric species or to *Ischnocnema.* In addition, karyotype differences, mainly in the NOR position, clearly distinguished the two *Barycholos* populations, besides indicating the existence of a so far undescribed species in this genus. A taxonomic review could clarify the systematic position of *P. crepitans* and verify the hypothetic new *Barycholos* species.

## Introduction

Recent taxonomic reviews based on molecular data (Frost *et al.*, 2006; Heinicke *et al.*, 2007; Hedges *et al.*, 2008) dramatically changed the long-standing systematics of the “eleutherodactyline” frogs (*sensu* Frost *et al.*, 2006). Heinicke *et al.* (2007) proposed four major clades for this anuran group, comprising the species from (1) the Caribbean (*Eleutherodactylus*), (2) Middle America (*Craugastor*), (3) Northern South America (*Pristimantis*), and (4) Southeastern Brazil (*Ischnocnema*), all of them placed in a single family named Brachycephalidae.

Based on DNA sequences from mitochondrial and nuclear genes of 344 species, Hedges *et al.* (2008) placed the 882 described species of Brachycephalidae into a new taxon, Terrarana, and classified them into four families, four subfamilies, 24 genera and 11 subgenera. Of those, two families, three subfamilies, six genera, and two subgenera were proposed and named as new taxa. The genera *Brachycephalus* and *Ischnocnema* remained in the Brachycephalidae family, a group restricted to the southeastern region of Brazil, as previously suggested by Heinicke *et al.* (2007). *Pristimantis* and *Barycholos* were allocated to the family Strabomantidae, subfamilies Strabomantinae e Holoadeninae, respectively, which are new taxa proposed by Hedges *et al.* (2008).

Thus far, more than 100 Terrarana species have been cytogenetically studied and reported as having a high degree of chromosome number variation, ranging from 2n = 18 to 36 (Bogart, 1991). Considering the arrangements proposed by Hedges *et al.* (2008), the *Craugastor* genus has 2n = 18, 20 and 22 chromosomes, *Diasporus* 2n = 18, *Brachycephalus* 2n = 22, *Ischnocnema* 2n = 20, 22 and 30, *Strabomantis* 2n = 20, 22, 34 and 35, while *Haddadus* and *Barycholos* have 2n = 22 chromosomes. The *Pristimantis* species display the greatest diploid complement variation, with 2n = 26, 30, 32, 34 and 36 chromosomes. Genus *Eleutherodactylus* (*sensu* Hedges *et al.*, 2008) is also highly variable, with 2n = 18, 22, 24, 26, 28, 30 and 32 chromosomes. Within the four *Eleutherodactylus* subgenera, *Eleutherodactylus* (*Eleutherodactylus*) have 2n = 18, 22, 26, 28 and 30, *Eleutherodactylus* (*Euhyas*) 2n = 24-32, *Eleutherodactylus* (*Pelorius*) 2n = 30, and *Eleutherodactylus* (*Syhrophus*) 2n = 22 and 30 (Duellman, 1967; Beçak, 1968; Brum-Zorrilla and Sáez, 1968; Bogart, 1970a,b,c, 1973, 1981, 1984, 1991; León, 1970; Beçak and Beçak, 1974; De Lucca and Jim, 1974; De Lucca *et al.*, 1974; DeWeese, 1975; Drewry and Jones, 1976; Savage and DeWeese, 1979, 1980; Green *et al.*, 1980; Miyamoto, 1983, 1984; Kaiser *et al.*, 1994, 1995; Bogart and Hedges, 1995; Savage and Myers, 2002; Siqueira *et al.*, 2004; Campos *et al.*, 2008). Additional cytogenetic studies on Terrarana species could help to improve the current taxonomic and evolutionary knowledge regarding this group.

In the present work, we analyzed two samples of *Ischnocnema juipoca*, two of *Barycholos* cf. *ternetzi* and two of *Pristimantis crepitans*. This latter species was not yet studied by molecular techniques, being included in genus *Pristimantis,* family Strabomantidae, based only on its geographic distribution (Hedges *et al.*, 2008). We aimed to increase the number of karyotyped Brazilian Terrarana species and further understand their taxonomy and evolutionary relatedness.

## Material and Methods

**Figure 1:**
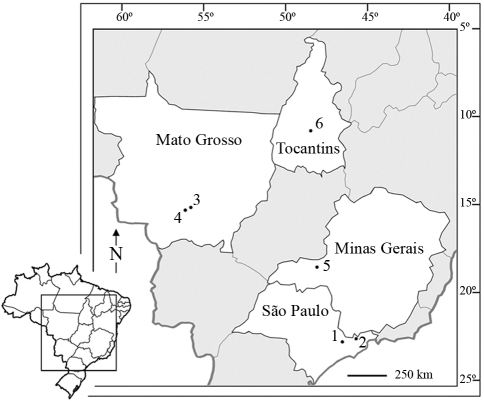
Map of Brazil showing the sampling sites where the studied frog populations were surveyed. 1: Atibaia, State of São Paulo, *Ischnocnema juipoca*; 2: Campos do Jordão, State of São Paulo, *I. juipoca*; 3: Chapada dos Guimarães, State of Mato Grosso, *Pristimantis crepitans*; 4: São Vicente, Cuiabá, State of Mato Grosso, *P. crepitans*; 5: Uberlândia, State of Minas Gerais, *Barycholos* cf. *ternetzi*; 6: Porto Nacional, State of Tocantins, *Barycholos* cf. *ternetzi*.

Specimens of *Ischnocnema juipoca,Barycholos* cf. *ternetzi* and *Pristimantis crepitans* were sampled under a permit (License nº 206/2005 - CGFAU/LIC) issued by IBAMA (Instituto Brasileiro do Meio Ambiente e dos Recursos Naturais Renováveis – Brazilian Institute of the Environment and Natural Renewable Resources). Voucher specimens were deposited in the Museu de Zoologia “Prof. Dr. Adão José Cardoso”, at the Universidade Estadual de Campinas (UNICAMP), Campinas, SP, Brazil, and in the Coleção “Célio F. B. Haddad” at the Universidade Estadual Paulista (UNESP), Rio Claro, SP, Brazil (Table 1). The sampling locations where the specimens were surveyed are displayed in Figure 1.

Mitotic chromosomes were obtained from suspensions of intestinal epithelium and testicular cells from animals pre-treated with 2% colchicine for at least 4 h, as described by King and Rofe (1976) and Schmid (1978). Conventional chromosome staining was performed with 10% Giemsa solution, Ag-NOR labeling (Howell and Black, 1980), and C-banding (Sumner, 1972), as modified by Siqueira *et al.* (2008). The slides were examined with a BX60 Olympus microscope and images were captured using the Image Pro-plus 4.5.1 and QCapture 2.81.0 softwares. Chromosomes were measured and classified according to Green and Sessions (1991).

## Results

**Figure 2:**
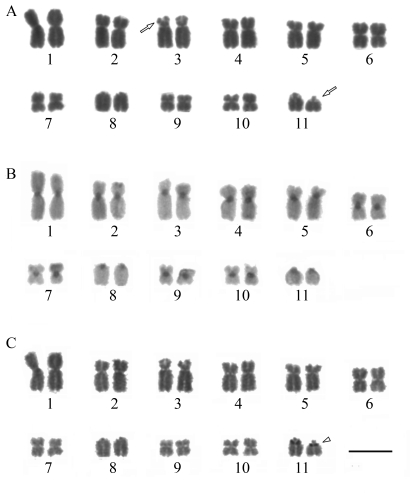
Karyotype of *Ischnocnema juipoca*: (**a**) Giemsa staining; (**b**) C-banding; (**c**) Ag-NOR labeling. The arrow indicates secondary constrictions.  The arrowheads indicate the NOR coincident with secondary constriction. Bar = 10 μm.

In all analyzed individuals, the diploid number was 22 chromosomes, but the Fundamental Numbers (FN) discriminated the three species. The FNs were determined as 40 in *I. juipoca,* 38 in *Barycholos* cf. *ternetzi*, and 44 in *Pristimantis crepitans* (Figures 2, -5).

### Ischnocnema juipoca

The karyotypes of the two analyzed *I. juipoca* populations consisted of five pairs of metacentric chromosomes (1, 6, 7, 9 and 10), four pairs of submetacentrics (2, 3, 4 and 5) and two pairs of telocentrics (8 and 11) (Figures 2A, -C). Secondary constrictions were present on the short arm of pair 3 and occasionally adjacent to the centromere of pair 11 (Figure 2A). Blocks of heterochromatin were detected in the centromeric region of all chromosomes, and in several metaphases there was a faint C-band adjacent to the centromere of pair 11, coinciding with the secondary constriction, and on the telomere of the short arm of pair 2 as well (Figure 2B). In the telocentric pair 11, the Ag-NOR sites were adjacent to the centromere and coincided with the secondary constriction and the pericentromeric block of heterochromatin (Figures 2A, -C).

Unusual size variation of the telocentric pairs was observed, both among metaphases of the same specimen and among different specimens. This size variation was probably due to differences in chromosome compaction and has hampered the positioning of these chromosomes in the karyogram, since they could be placed in any position among the last four pairs. Size variation of the telocentric chromosomes was also observed in the *Barycholos* populations.

### *Barycholos* cf. *ternetzi* (Uberlândia, MG)

**Figure 3:**
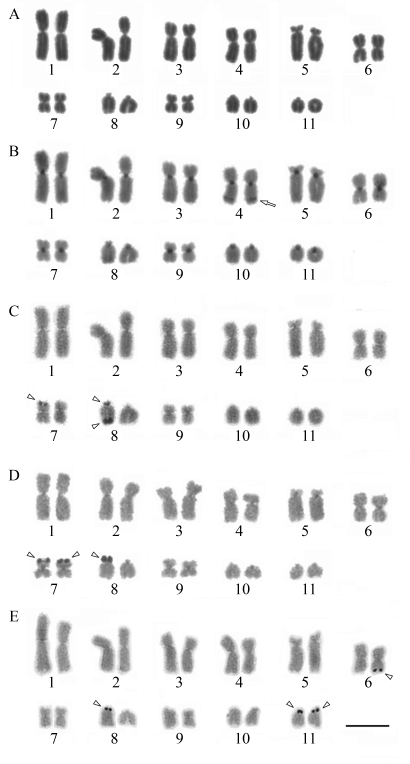
Karyotype of *Barycholos* cf. *ternetzi* (Uberlândia, MG): (**a**) Giemsa staining; (**b**) C-banding; (**c-e**) Ag-NOR staining. The arrow indicates interstitial heterochromatin. The arrowheads indicate NORs. Note the distinct position and number of NORs in **c, d** and **e**. Bar = 10 μm.

The karyotype of *Barycholos* cf. *ternetzi* consisted of six metacentric (1, 2, 4, 6, 7 and 9), one submetacentric (pair 3), one subtelocentric (pair 5) and three telocentric (8, 10 and 11) pairs (Figures 3A, -E). In several metaphases, secondary constrictions were found on the telomere of the NOR-carrying telocentric chromosome 8 (Figures 3A, -E). Three distinct NOR patterns were detected: (1) in three specimens, three Ag-NOR sites were observed on the telomere of one pair 7 homologue, and two on one pair 8 homologue, one on the centromere and the other on the telomere (Figure 3C); (2) in four specimens, three Ag-NOR sites were found, two on the telomeres of pair 7 and one adjacent to the centromere of one chromosome of pair 8 (Figure 3D); and (3) in two specimens, four Ag-NORs were found, one on the telomere of one pair 6 homologue and one on the centromere of one pair 8 homologue, and two labels on the centromeres of pair 11 (Figure 3E). Heterochromatic blocks were detected in the centromeric region of all chromosomes, and a faint C-band was observed near the telomere on the long arm of pair 4 (Figure 3B).

### *Barycholos* cf. *ternetzi* (Porto Nacional, TO)

**Figure 4:**
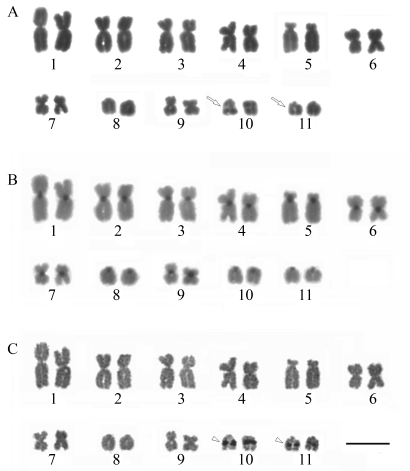
Karyotype of *Barycholos* cf. *ternetzi* (Porto Nacional, TO): (**a**) Giemsa staining; (**b**) C-banding; (**c**) Ag-NOR staining. The arrow indicates secondary constrictions. The arrowheads indicate the NORs coincident with secondary constriction. Bar = 10 μm.

The *Barycholos* cf. *ternetzi* karyotype consisted of six metacentric (1, 2, 4, 6, 7 and 9), one submetacentric (pair 3), one subtelocentric (pair 5) and three telocentric (8, 10 and 11) pairs (Figures 4A, -C). Secondary constrictions were found interstitially on pairs 10 and 11 (Figures 4A, -C). The Ag-NORs were interstitially located in pairs 10 and 11, coincident with the secondary constrictions (Figure 4C). The NORs were heteromorphic in pair 11. Heterochromatic blocks were limited to the centromeric region of all chromosomes (Figure 4B).

### Pristimantis crepitans

**Figure 5:**
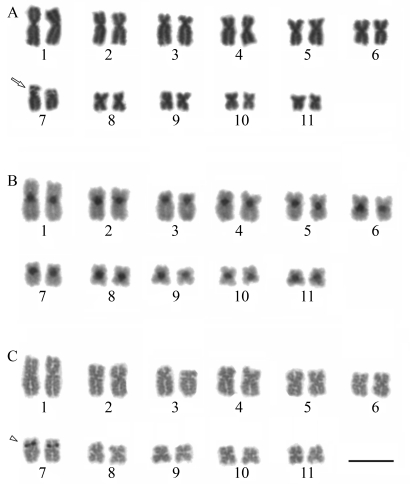
Karyotype of *Pristimantis crepitans*: (**a**) Giemsa staining; (**b**) C-banding; (**c**) Ag-NOR staining. The arrow indicates the secondary constriction. The arrowheads indicate the NORs coincident with secondary constrictions. Bar = 10 μm.

The *P. crepitans* karyotype consisted of eigth pairs of metacentric (1, 2, 5, 6, 8, 9, 10 and 11) and three of submetacentric chromosomes (3, 4 and 7). Interstitial secondary constrictions were observed on the long arms of pair 7, where the NOR sites were detected (Figures 5A and C). Blocks of strongly stained heterochromatin were located in the centromeric region of all chromosomes (Figure 5B).

## Discussion

The diploid number of 22 chromosomes observed in *I. juipoca, Barycholos* cf. *ternetzi* and *P. crepitans* has also been described for 28 other Terrarana frog species*.* In this anuran group, *P. crepitans* is the only species with such a low chromosome number occurring in the midwest of Brazil. The species with 2n = 20 and 22 are typically distributed in southeastern and southern Brazil, while most Brazilian species of the northern and northeastern regions have 2n = 30 and 34 (Bogart, 1973; DeWeese, 1975; Siqueira *et al.*, 2004, Siqueira *et al.*, 2008).

The chromosome morphology and C-banding patterns found in the karyotype of the *Barycholos* cf. *ternetzi* specimens from Uberlândia were very similar to those previously described in Gurinhatã specimens (Campos *et al.*, 2008). These two sampling sites are located in the State of Minas Gerais, not very distant from each other. Some small karyotype differences may have resulted from the use of different chromosome preparation techniques and from the classification methods used for karyotype description. Moreover, in the *Barycholos* specimens from Uberlândia, the only heterochromatic block detected on the long arm of pair 4 corresponded to a band on pair 4 of the C-banded karyotype of *Barycholos ternetzi* (Gurinhatã), as shown by Campos *et al.* (2008), although the authors did not explicitly mention this band. In contrast, the karyotype of *Barycholos* cf. *ternetzi* from Uberlândia showed variation in number and position of NORs, as also reported for *B. ternetzi* from Gurinhatã by Campos *et al.* (2008). These authors suggested that the fixed NOR must be that on pair 11. In the present work, we found three additional NOR patterns. Of these, only one was observed on pair 11, indicating that the principal NOR-bearing chromosome is still uncertain.

The variation in number and position of NOR-labeling in the two studied *Barycholos* populations, one by Campos *et al.* (2008) and the *Barycholos* cf. *ternetzi* presented herein, suggests the occurrence of chromosomal rearrangements involving mainly the telomeric regions. In anuran species, transposition of mobile genetic elements, ribosomal cistron amplification, and rDNA reinsertion errors during extra chromosomal amplification of ribosomal cistrons have been suggested as responsible for such NOR variations (Wiley *et al.*, 1989; King *et al.*, 1990; Foote *et al.*, 1991; Schmid *et al.*, 1995; Kaiser, 1996; Lourenço *et al.*, 1998). The variation in NOR number and location may represent an incipient process of species differentiation, and a taxonomic review, including additional methodologies, could clarify if this is indeed a new taxon.

Regarding the *Barycholos* cf. *ternetzi* specimens from Porto Nacional, the Ag-NOR labeling was fixed in two chromosome pairs (10 and 11) and located in an interstitial position, differently from the telomeric position observed in the *Barycholos* cf. *ternetzi* specimens from Uberlândia. The four analyzed *Barycholos* cf. ternetzi specimens from Porto Nacional also differed from the *B. ternetzi* from Gurinhatã (Campos *et al.*, 2008). Additionally, the specimens from Porto Nacional did not show the heterochromatic block on the long arm of pair 4.

In spite of the similar chromosome morphology presented by the two *Barycholos* cf. *ternetzi* populations studied, the karyotypic differences*,* mainly in NOR position and C-banding, indicate the possible existence of two species and call attention to the need of a taxonomic review of these populations. The NOR locations and heterochromatin pattern have been useful to distinguish among different species such as, for example, *Scythrophrys* (Lourenço *et al.*, 2003a, 2008), *Paratelmatobius* (Lourenço *et al.*, 2003b*,* 2008) and *Pristimantis dundeei* and *Pristimantis.* aff*. dundeei* (Siqueira *et al.*, 2008). Moreover, since vocalization and chromosomal features of the topotypical population are unknown, none of the already studied populations can be assigned with certainty to the nominal *Barycholos ternetzi*.

The data obtained for *I.juipoca* from Atibaia and Campos do Jordão are in agreement with those described by Campos *et al.* (2008) for populations from Itatiba and Santa Branca, also in the State of São Paulo. The other karyotyped species of genera *Ischnocnema* and *Brachycephalus,* both within family Brachycephalidae, were substantially diverse from those described herein. Those other species, such as *I. guentheri,I. parva* and *B. ephippium*, have the same chromosome number as *I. juipoca* (2n = 22), but no telocentric chromosomes (Siqueira *et al.*, 2004; Ananias *et al.*, 2006), whereas *I. holti* and *I. lactea* present a diploid number of 20 chromosomes (De Lucca and Jim, 1974; De Lucca *et al.*, 1974). In addition, in *B. ephippium* the NOR was located interstitially on the metacentric pair 8, while in *I. juipoca* it was on the telocentric pair 11.

*Pristimantis crepitans* was removed from the synonymy of *Eleutherodactylus fenestratus*, (Heyer and Muñoz, 1999), where it was originally placed (Lynch, 1980). Hedges *et al.* (2008) allocated *P. crepitans* to the *P. peruvianus g*roup, and J. M. Padial (pers. comm. to Hedges *et al.*, 2008) indicated the possibility that both *P. crepitans* and *P. dundeei* might belong to the *P. conspicillatus* group. However, the low chromosome number and the ecological characteristics of *P. crepitans* and *P. dundeei* indicate that these species are not closely related to the other *Pristimantis.* The putative taxonomic position of the former within genus *Pristimantis*, as proposed by Heinicke *et al.* (2007) and Hedges *et al.* (2008), was based solely on its geographical distribution, since it was never included in any molecular analysis. The low chromosome number (2n = 22) of *P. crepitans* is commonly found in *Ischnocnema* and is highly divergent from other known *Pristimantis* karyotypes, which typically have high chromosome numbers (2n = 30 to 34). Unfortunately, the only other *Pristimantis* species with a low diploid number, *P*. *altae* with 2n = 26 (DeWeese, 1975), was not yet submitted to molecular analysis. On the other hand, a preliminary analysis of spermatozoa ultrastructure indicated great differences between *P. crepitans* and the other *Pristimantis* species, as well as the studied *Ischnocnema* species (S. Siqueira S., unpublished data)*.Pristimantiscrepitans* is also unique by living in open and xeric habitats in the Cerrado biome, among granitic or arenitic outcrops. These divergences strongly suggest a need of complementary molecular analysis to reassess the recently proposed allocation of *P. crepitans* to genus *Pristimantis* (Heinicke *et al.*, 2007; Hedges *et al.*, 2008). Therefore, further studies are necessary to clarify the systematic position of these species.

## Concluding Remarks

Major contributions to the understanding of the molecular phylogeny of the South American eleutherodactyline species were recently brought by Frost *et al.* (2006), Heinicke *et al.* (2007) and Hedges *et al.* (2008). However, for Brazilian species there are many unresolved taxonomic aspects which require further investigation using a combination of morphological, cytogenetic and molecular techniques. Based on previously reported molecular data (Frost *et al.*, 2006), behavioral studies (Caramaschi and Pombal, 2001), and karyotypes (Campos *et al.*, 2008) it seems conceivable that *Barycholos* and the already karyotyped *Ischnocnema* species are close relatives*.* However, the additional molecular studies of Hedges *et al.* (2008) indicate that *Barycholos* is phylogenetically distant from both *Ischnocnema* and *Haddadus binotatus.* Therefore, the observed chromosomal similarities might be symplesiomorphies or the result of convergence generated by chromosomal rearrangements, thus not substantiating a hypothesis of close evolutionary relationships between the species *I. juipoca* and *Barycholos.*

In the new systematic arrangement for “eleutherodactyline” frogs proposed by Heinicke *et al.* (2007) and Hedges *et al.* (2008), mostly based on molecular data, there are still indications of divergences in relation to the available karyological data. Most likely, the divergences are due to the lack of molecular analysis of many of the Brazilian karyotyped species, such as *Pristimantis crepitans* and *P. altae*. Further molecular and chromosome analyses of Terrarana frogs, including these divergent species, should provide a broader understanding of their evolutionary relatedness and systematic status.

## Figures and Tables

**Table 1:** Voucher specimens, sampling sites and accession numbers.

Species	Male (n)	Female (n)	Sampling site	ZUEC and CFBH* accession numbers
*Ischnocnema juipoca*	2	1	Atibaia and Campos do Jordão, State of São Paulo	13265, 13266, 9904*
*Pristimantis crepitans*	3	2	Chapada dos Guimarães and Distrito de São Vicente, Cuiabá, State of Mato Grosso	14114-14119
*Barycholos* cf. *ternetzi*	7	2	Uberlândia, State of Minas Gerais	13262-13264, 13475, 13476, 14120-14123
*Barycholos* cf. *ternetzi*	-	4	Porto Nacional, State of Tocantins	14124-14127

n = number of specimens analyzed; accession numbers at the Museu de Zoologia “Prof. Adão José Cardoso” (ZUEC), UNICAMP.
